# Molecular Characterization of Prothionamide-Resistant *Mycobacterium tuberculosis* Isolates in Southern China

**DOI:** 10.3389/fmicb.2017.02358

**Published:** 2017-11-30

**Authors:** Yaoju Tan, Biyi Su, Huiwen Zheng, Yuanyuan Song, Yufeng Wang, Yu Pang

**Affiliations:** ^1^Department of Clinical Laboratory, Guangzhou Chest Hospital, Guangzhou, China; ^2^National Clinical Laboratory on Tuberculosis, Beijing Key Laboratory on Drug-resistant Tuberculosis Research, Beijing Chest Hospital, Capital Medical University, Beijing Tuberculosis and Thoracic Tumor Institute, Beijing, China; ^3^National Center for Tuberculosis Control and Prevention, Chinese Center for Disease Control and Prevention, Beijing, China

**Keywords:** *Mycobacterium tuberculosis*, prothionamide, genetic mutation, resistance, Beijing genotype

## Abstract

Prothionamide (PTH) has been widely used in the treatment of tuberculosis (TB), especially multidrug resistant tuberculosis (MDR-TB), while data regarding prevalence of resistance-causing mutation is limited. In this study, we aimed to investigate the molecular characteristics of PTH-resistant MTB isolates, and also analyzed the risk factors for PTH resistance among *Mycobacterium tuberculosis* (MTB) isolates in southern China. A total of 282 MTB isolates were enrolled in from Guangzhou Chest Hospital. Among these isolates, 46 (16.3%) were resistant to PTH. Statistical analysis revealed that PTH resistance was more likely to be associated with resistance to levofloxacin (LFX; OR: 2.18, 95% CI: 1.02–4.63; *P* = 0.04). Of the 46 PTH-resistant MTB isolates, 37 (80.4%) isolates harbored 19 different mutation types, including 10 (21.7%) isolates with double nucleotide substitutions and 27 (58.7%) with single nucleotide substitution. The mutations in ethA (51.4%, 19/37) were most frequently observed among PTH-resistant isolates, followed by 16 (43.2%) in the promoter of inhA and 6 (16.2%) in inhA. In addition, no significant difference was found in the distribution of isolates with different mutation types between Beijing and non-Beijing genotypes (*P* > 0.05). In conclusion, our data demonstrate that high diversity of genetic mutations conferring PTH resistance is identified among MTB isolates from southern China. Mutations in inhA, ethA, mshA, and ndh genes confer increased resistance of MTB to PTH. Ancient Beijing genotype strains have higher proportion of drug resistance compared with modern Beijing strains. In addition, PTH resistance is more likely to be observed in the LFX-resistant MTB isolates.

## Introduction

Tuberculosis (TB), caused by *Mycobacterium tuberculosis* complex (MTBC), continues to be a major cause of morbidity and mortality worldwide, with estimated 9.6 million new cases and 1.5 million deaths reported in 2015 (WHO, 2016). Despite achieving a huge effort to curb the TB epidemic in the last two decades, the emergency of drug-resistant TB, especially multidrug-resistant TB (MDR-TB) and extensively drug-resistant tuberculosis (XDR-TB), have highlighted the urgent need to address TB more effectively on a global scale (Zhao et al., 2012).

Prothionamide (PTH), a member of thioamides, has been widely used for many years in the treatment of MDR-TB, as well as drug susceptible tuberculous meningitis and miliary TB in several settings (Wang et al., 2007; Thee et al., 2016). *In vitro* studies show that PTH has significant bactericidal effect against MTB (Heifets et al., 1991; Thee et al., 2016). Compared with the ethionamide (ETH), another member of thioamides, PTH might yield lower prevalence of adverse effects and be better tolerated, which indicates its promising perspective for future clinical applications (Fox et al., 1969; Gupta et al., 1977). Similar to INH, PTH is also a prodrug that requires *in vivo* activation to form adducts with NAD to subsequently block the action of fatty acid synthase (Wang et al., 2007). Notably, *katG*-encoded catalase peroxidase involves the activation of INH to form the INH-NAD adduct, while PTH employs *ethA*—encoded monooxygenase to catalyze the transition of PTH-NAD adduct (Vilchèze et al., 2008). Hence, the genetic mutations in the *ethA* gene serve as the most important mechanism conferring PTH resistant in MTB. As the target of PTH, mutations at the promoter region of *inhA* gene cause the overexpression or modification of the InhA, thereby resulting in PTH resistance (Rueda et al., 2015). Recently, several novel genes have also been identified to be attributed to the decrease in susceptibility to thioamides in MTB, including *ethR, mshA*, and *ndh* (DeBarber et al., 2000; Morlock et al., 2003; Vilchèze et al., 2005, 2008; Hazbón et al., 2006).

Accurate molecular diagnosis of drug-resistant TB is dependent on the knowledge of the mechanisms of resistance to anti-TB drugs, as well as the prevalence of resistance-causing mutation (Green and Garneau-Tsodikova, 2013). Although several genes conferring thioamide resistance have been reported, more attention has been focused on ETH rather than PTH in the past decades (Thee et al., 2016). The results from previous studies on mechanism of drug resistance to ETH become a major source of knowledge regarding this aspect of PTH resistance in MTB given that the two drugs display a high degree of cross-resistance (Projahn et al., 2011). Hence, it is meaningful to investigate the molecular characteristics of PTH-resistant MTB isolates (Projahn et al., 2011). In this study, we firstly carried out a molecular epidemiological study on this issue in China. A total of 10 candidate genes contributing to PTH resistance were enrolled in this study, including seven reported gene (inhA, the promoter of inhA, ethA, ethR, ndh, folC, mshA), and three genes conferring mycothiol biosynthesis (mshB, mshC, and mshD), which has been documented to be essential for ethionamide susceptibility in MTB (Vilchèze et al., 2008). In addition, we analyzed the risk factors for PTH resistance, and also investigate the relationship between PTH resistance and MTB genotypes in southern China.

## Materials and methods

### Ethical statement

This study was approved by the Ethics Committee of Guangzhou Chest Hospital (GZXK-2016-012). The patients were enrolled in this study after providing the written consent by themselves.

### Bacterial strains

A total of 282 MTB clinical isolates from TB patients were enrolled from the largest TB specialized hospital of southern China, Guangzhou Chest Hospital, between January 2016 and June 2016. The demographic characteristics were obtained from the electronical medical record. Two sputum samples were collected from each participant for mycobacterial culture with BD BancTec MGIT 960. The positive culture was subcultured on the Löwenstein-Jensen (L-J) medium for drug susceptibility testing (DST) and DNA extraction. Prior to DST, species identification was performed using a commercial MPB64 monoclonal antibody assay (Genesis, Hangzhou, China) (Pang et al., 2016).

### Phenotypical drug susceptibility testing

The BACTEC MGIT 960 system was used to detect the drug susceptibility of MTB isolates against first-line and second-line drugs (Zhang et al., 2014b). Briefly, the cell suspensions of *M. tuberculosis* strains were adjusted to a 0.5 McFarland standard with sterile normal saline. Then the standardized suspensions were diluted 1:5 with sterile normal saline for inoculation into PTH-containing MGIT tubes. For inoculation of the growth control tube, the 1:5 cell suspensions were further diluted into 100-fold with sterile normal saline. 0.5 ml of each inoculum was added in the corresponding MGIT tubes. The drug resistance was defined as the growth unit (GU) values of PTH-containing tubes were more than 100, when the GU-values of growth control tube reached a GU-value of 400. According to the recommendation from WHO, the concentrations of drugs in media were as follows: isoniazid (INH) 0.1 μg/mL, rifampin (RIF) 2.0 μg/mL, ethambutol (EMB) 2.5 μg/ml, streptomycin (SM) 2.0 μg/mL, amikacin (AMK) 1.0 μg/mL, levofloxacin (LFX) 2.0 μg/mL, and PTH 2.5 μg/mL (WHO, 2008). The clinical laboratory of Guangzhou Chest Hospital has passed the annual proficiency testing for phenotypical DST organized by the National TB Reference Laboratory of the China CDC. MDR-TB is defined as TB resistant to at least INH and RIF, and XDR-TB is defined as MDR-TB plus resistance to any fluoroquinolone (FQ) and at least one second-line anti-TB injectable drug.

### PCR amplification and sequencing

The crude genomic DNA was extracted from freshly bacteria as previously described (Pang et al., 2012). The cultured mycobacteria was harvested from the surface of L-J medium, and then transferred into a microcentrifuge tube with 500 μL Tris-EDTA (TE) buffer. The tube was heated in a 95°C water bath for 1 h. Followed by centrifugation at 13,000 rpm for 2 min, the supernatant was pipetted for polymerase chain reaction (PCR) amplification. The fragments of putative genes conferring PTH resistance, including *inhA*, the promoter of *inhA, ethA, ethR, ndh, folC, mshA, mshB, mshC*, and *mshD* were amplified with primers listed in Table 1. The PCR reaction mixture was prepared as follows: 25 μL of 2 × PCR Mixture [containing 20 mM Tris-HCl (pH 8.3), 100 mM KCl, 3 mM MgCl_2_, 400 μM of each deoxynucleotide triphosphate, and 5 U of Taq polymerase] (Genestar, Beijing, China), 3 μL of DNA template and 0.2 μM of each primer set. PCR program consisted of an initial denaturation at 94°C for 5 min, followed by 35 cycles of denaturation at 94°C for 1 min, annealing at 58°C for 1 min, and extension at 72°C for 2 min, and a final extension at 72°C for 10 min. PCR products were analyzed by electrophoresis on 1.5% agarose gel (Supplemental Figure 1), and then purified with a Magbind PCR Purification Kit (CWBio, Beijing, China), and then sent to Qingke Company for DNA bidirectional sequencing service. DNA sequences were aligned with the homologous sequences of MTB standard H37Rv strain (GenBank accession number NC_000962). The Gene ID of each sequence used for designing primers and aligning sequencing chromatograms is as follows: *inhA*, Gene ID 886523; *ethA*, Gene ID 886175; *ethR*, Gene ID 886189; *ndh*, Gene ID 885746; *folC*, Gene ID: 885902; *mshA*, Gene ID 887160; *mshB*, Gene ID 885997; *mshC*, Gene ID 887492 and *mshD*, Gene ID 885251. The DNA sequences of *ethA, ndh, mshA* with nucleotide substitutions in this paper have been submitted to GenBank, with accession numbers MG182066-MG18073 and MG182052-182056 for *ethA*; MG182059-MG182065 for *ndh*, MG182057-MG182058 for *mshA*, respectively.

**Table 1:** Candidate genes conferring PTH resistance in *Mycobacterium tuberculosis*.

**Locus**	**Alias**	**Annotation**	**PCR primer pairs^a^**
*inhA*	Rv1484	NADH-dependent enoyl-[ACP] reductase	F 5′-GCAGCATGCAGCGCA ACA AATTC-3′
			R 5′-GTAGGGCCGCAGTTTTACCAGTTC−3′
*inhA promoter*	–	–	F 5′-CCTCGCTGCCCAGAA AGGGA
			R 5′-ATCCCCCGGTTTCCTCCGGT
*ethA*	Rv3854c	Monooxygenase	F 5′-CCTGGCAGCTTACTACGTGTC-3′
			R 5′-CGGCATCATCGTCGTCTG-3′
*ethR*	Rv3855	TetR family transcriptional regulator	F 5′-TTTTCCAGGATGGCGTAGC-3 ′
			R 5′-CCGACCGGATCGTCAACA-3′
*ndh*	Rv1854c	NADH dehydrogenase	F 5′-ACTTGGCTCCGCACGGCTAT-3′
			R 5′-ATCCGGCGACGGCATTCA-3′
*mshA*	Rv0486	D-inositol 3-phosphate glycosyltransferase	F 5′-CCGCTACCGCCATCACCGACTT-3′
			R 5′-GGCCGCACGCAGCACAAT-3′
*mshB*	Rv1170	1D-myo-inositol 2-acetamido-2-deoxy-alpha-D-glucopyranoside deacetylase	F 5′-TTTTCACCATAACGGCTTCGG-3′
			R 5′-AGGCTGTCGAGTCGGTCCTG-3′
*mshC*	Rv2130c	Cysteine:1D-myo-inosityl 2-amino-2-deoxy–D-glucopyranoside ligase	F 5′-TCAGAAAGGCTGGAGTCACCG-3′
			R 5′-GCGATGTCATGGCCGAAA-3′
*mshD*	Rv0819	Mycothiol acetyltransferase	F 5′-CCGGGATGGTATGCAAGAAC-3′
			R 5′-CCGCATGAAATGACCCGTAG-3′
*folC*	Rv2447c	Folylpolyglutamate synthase	F 5′-TGAGCATCTACTCGACCAACGC-3′
			R 5′-GCGGATCACGACCGAACAAG-3′

a *F, forward; R, reverse*.

### Genotyping

The RD105 deletion-targeted multiplex PCR (DTM-PCR) was used to differentiate Beijing genotype and non-Beijing genotype according to the previous literature (Zhang et al., 2015). The strains without amplification of RD105 region were classified to Beijing genotype, while the others harboring RD105 region belonged to non-Beijing genotype. Further identification of the IS6110 PCR in the NTF region was performed to distinguish modern Beijing from ancient Beijing strains as previously reported (Zhang et al., 2015). The strains with a 1.8-kb DNA fragment in the NTF region were categorized as modern Beijing genotype, while the others with a 700-bp insertion in the NTF region were ancient Beijing genotype.

### Statistical analysis

Associations among multiple categorical variables between PTH-resistant and PTH-susceptible groups were firstly analyzed with Pearson Chi-square test. All variables with a *P* < 0.05 in univariate analysis were further included in the forward stepwise logistic regression procedures to determine the independent covariates associated with PTH resistance. Differences were declared as statistically significant if a *P*-value was less than 0.05. All statistical analysis was calculated with SPSS 11.5 (SPSS Inc., USA).

## Results

### Demographic characteristics

Of the 282 MTB isolates, 200 (70.9%) were from male patients and 82 (29.1%) were from female patients. Age-wise analysis of MTB isolates showed that all the isolates belonged to patients aged 18–86 years, with a mean age of 41.2 years. In addition, the majority (79.1%, 223/282) of TB patients enrolled in this study was migrant population, and 40.8% (115/282) were new cases (Table 2).

**Table 2:** Univariate analysis of factors associated with PTH resistance.

**Characteristics**	**No. of isolates**		**OR 95%CI**	***P*-value**
	**PTH-R (*n* = 46)**	**PTH-S (*n* = 236)**		
**GENDER**				
Male	38 (82.6)	162 (68.6)	2.17 (0.97–4.88)	0.06
Female	8 (17.4)	74 (31.4)	1.00 (Ref.)	–
**AGE GROUP (YEARS)**				
<25	9 (19.6)	47 (19.9)	1.01 (0.41–2.52)	0.98
25–44	14 (30.4)	74 (31.3)	1.00 (Ref.)	–
45–65	18 (39.1)	85 (36.0)	1.12 (0.52–2.40)	0.77
>65	5 (10.9)	30 (12.7)	0.88 (0.29–2.66)	0.82
**POPULATION**				
Resisdent	9 (19.6)	50 (21.2)	1.00 (Ref.)	–
Migrant	37 (80.4)	186 (78.8)	1.10 (0.50–2.44)	0.81
**TREATMENT HISTORY**				
New case	14 (30.4)	101 (42.8)	1.00 (Ref.)	–
Retreated	32 (69.6)	135 (57.2)	1.71 (0.87–3.37)	0.12
**RIF**				
Yes	20 (43.5)	55 (23.3)	2.53 (1.31–4.88)	0.01
No	26 (56.5)	181 (76.7)	1.00 (Ref.)	–
**INH**				
Yes	28 (60.9)	114 (48.3)	1.66 (0.87–3.17)	0.12
No	18 (39.1)	122 (51.7)	1.00 (Ref.)	–
**SM**				
Yes	24 (52.2)	79 (33.5)	2.17 (1.14–4.11)	0.02
No	22 (47.8)	157 (66.5)	1.00 (Ref.)	–
**EMB**				
Yes	14 (30.4)	45 (19.1)	1.86 (0.92–3.77)	0.08
No	32 (69.6)	191 (80.9)	1.00 (Ref.)	–
**AMK**				
Yes	4 (8.7)	11 (4.7)	1.95 (0.59-6.41)	0.27
No	42 (91.3)	225 (95.3)	1.00 (Ref.)	–
**LFX**				
Yes	15 (47.8)	36 (15.3)	2.69 (1.32–5.48)	< 0.01
No	31 (52.2)	200 (84.7)	1.00 (Ref.)	–
**MDR–TB**				
Yes	18 (39.1)	54 (22.9)	2.17 (1.11–4.21)	0.02
No	28 (60.9)	182 (77.1)	1.00 (Ref.)	–

*RIF, rifampin; INH, isoniazid; SM, streptomycin; EMB, ethambutol; AMK, amikacin; LFX, levofloxacin; PTO, prothionamide; MDR-TB, multidrug resistant tuberculosis; OR, odds ratio; 95% CI: 95% confidence interval.

### Factors associated with PTH resistance

Among the 282 MTB isolates, 75 (26.6%) were resistant to RIF, 142 (50.4%) to INH, 103 (36.5%) to SM, 59 (20.9%) to EMB, 15 (5.3%) to AMK, 51 (18.1%) to LFX, and 46 (16.3%) to PTH. In addition, 72 (25.5%) and 12 (4.3%) isolates were identified as MDR-TB and XDR-TB, respectively. We further analyzed the factor associated with PTH resistance. As shown in Table 2, statistical analysis revealed that PTH resistance was more likely to be associated with resistance to RIF [odds ratio (OR): 2.53, 95% confidence interval (95%CI): 1.31–4.88; *P* = 0.01], SM (OR: 2.17, 95% CI: 1.14–4.11; *P* = 0.02), LFX (OR: 2.69, 95% CI: 1.32–5.48; *P* < 0.01) and MDR-TB (OR: 2.17, 95% CI: 1.11–4.21; *P* = 0.02), whereas there was no significant difference in the distribution of PTH-resistant MTB isolates according to gender, age, residence and treatment history (*P* > 0.05; Table 2). In the multivariate analysis, TB patients with LFX resistance demonstrated a significantly increased risk for developing PTH resistance, with an OR of 2.18 (95% CI: 1.02–4.63, *P* = 0.04; Table 3).

**Table 3:** Multivariable analysis for factors associated with PTH resistance.

**Characteristics**	**PTH-resistant vs. PTH-susceptible**	
	**Adjusted OR (95%CI)**	***P*-value**
RIF resistance	1.50 (0.62–3.66)	0.37
SM resistance	1.76 (0.89–3.47)	0.10
LFX resistance	2.18 (1.02–4.63)	0.04

*RIF, rifampin; SM, streptomycin; LFX, levofloxacin; OR, odd ratio; 95% CI: 95% confidence interval.

### Association between beijing genotype and drug resistance

Genotyping data of the MTB isolates showed that 218 strains (77.3%) belonged to Beijing genotype, and the other 64 (22.7%) to non-Beijing genotype. Out of 218 Beijing genotype strains, there were 151 (69.3%, 151/218) and 67 (30.7%, 67/218) strains identified as modern and ancient Beijing genotype, respectively. As shown in Table 4, the percentages of INH-resistant, SM-resistant, EMB-resistant, and MDR TB in Beijing genotype were significantly higher than non-Beijing genotype (54.6 vs. 35.9% for INH; 39.9 vs. 25.0% for SM; 23.9 vs. 10.9% for EMB; 28.4 vs. 15.6% for MDR, *P* < 0.05), whereas the percentages of the other drug susceptibility profiles showed no difference between Beijing genotype and non-Beijing Beijing genotype. We also compared the percentages of drug-resistance between TB patients identified as infected with ancient Beijing genotype and modern Beijing genotype. Our data demonstrated that the proportions of INH-resistance and SM-resistance was significantly lower in the modern Beijing genotype than in the ancient Beijing genotype group (68.7 vs. 48.3% for INH; 50.7 vs. 35.1% for SM, *P* < 0.05). In addition, no difference was observed in PTH resistance between Beijing genotype and non-Beijing genotype (*P* = 0.55), also between modern and ancient Beijing genotype (*P* = 0.53).

**Table 4:** Distribution of drug resistant MTB isolates among Beijing and non-Beijing genotypes.

**Drug**	**No. of drug resistant isolates (%)**						
	**Beijing (*****n*** = **218)**				**Non-Beijing (*n* = 64)**	***P*-value**	**Total (*n* = 282)**
	**Ancient (*n* = 67)**	**Modern (*n* = 151)**	***P*-value**	**Total**			
RIF	22 (32.8)	42 (27.8)	0.45	64 (29.4)	11 (17.2)	0.05	75 (26.6)
INH	46 (68.7)	73 (48.3)	0.01	119 (54.6)	23 (35.9)	0.01	142 (50.4)
SM	34 (50.7)	53 (35.1)	0.03	87 (39.9)	16 (25.0)	0.03	103 (36.5)
EMB	19 (28.4)	33 (21.9)	0.30	52 (23.9)	7 (10.9)	0.03	59 (20.9)
AMK	4 (6.0)	9 (6.0)	1.00	13 (6.0)	2 (3.1)	0.37	15 (5.3)
LFX	17 (25.4)	25 (16.6)	0.13	42 (19.3)	9 (14.1)	0.34	51 (18.1)
PTH	12 (17.9)	22 (14.9)	0.53	34 (15.6)	12 (18.8)	0.55	46 (16.3)
MDR-TB	22 (32.8)	40 (26.5)	0.34	62 (28.4)	10 (15.6)	0.04	72 (25.5)
XDR-TB	3 (4.5)	7 (4.6)	1.00	10 (4.6)	2 (3.1)	1.00	12 (4.3)

*RIF, rifampin; INH, isoniazid; SM, streptomycin; EMB, ethambutol; AMK, amikacin; LFX, levofloxacin; PTH, prothionamide; MDR-TB, multidrug resistant tuberculosis; XDR-TB, extensively drug resistant tuberculosis.

### Mutations identified in PTH-resistant MTB isolates

The 10 candidate genes contributing to PTH resistance were firstly sequenced in 46 PTH-resistant MTB isolates. Descriptions of non-synonymous mutations conferring PTH resistance observed in this study were summarized in Table 5. All the mutations were located in five genes from the PTH-resistant MTB isolates, whereas no genetic substitutions were detected among other five genes, including folC, ethR, mshB, mshC, and mshD. Of the 46 PTH-resistant MTB isolates, 37 (80.4%) isolates harbored 19 different mutation types, including 10 (21.7%) isolates with double nucleotide substitutions and 27 (58.7%) with single nucleotide substitution. The mutations in ethA (51.4%, 19/37) were most frequently observed among PTH-resistant isolates, followed by 16 (43.2%) in the promoter of *inhA*, 6 (16.2%) in *inhA*, 4 (10.8%) in *ndh* and 2 (5.4%) in *mshA*, respectively. For the *ethA* gene, the mutation located in codon 266 (7/37, Ser266Arg) was identified as the most prevalent mutation related to PTH resistance.

**Table 5:** Mutations in *inhA, ethA, mshA*, and *ndh* in 46 PTH-resistant MTB isolates.

**No. of isolates (%)**	**Resistance genotype**				
	***inhA promoter***	***inhA ORF***	***ethA***	***mshA***	***ndh***
5 (10.9)	WT	WT	S266R (AGC → AGG)	WT	WT
4 (8.7)	−15C → T	S94A	WT	WT	WT
4 (8.7)	−8T → C	WT	WT	WT	WT
4 (8.7)	WT	WT	E36Q (GAA → CAA)	WT	WT
3 (6.5)	−15C → T	WT	WT	WT	WT
2 (4.3)	WT	S94A	WT	WT	WT
2 (4.3)	−15C → T	WT	E36Q (GAA → CAA)	WT	WT
2 (4.3)	WT	WT	S266R (AGC → AGG)	WT	WT
1 (2.2)	WT	WT	W69R (TGG → CGG)	WT	WT
1 (2.2)	WT	WT	R227P (CGC → CCC)	WT	WT
1 (2.2)	−15C → T	WT	T314P (ACC → CCC)	WT	WT
1 (2.2)	WT	WT	T392R (ACG → AGG)	WT	WT
1 (2.2)	WT	WT	L301R (CTG → CGG)	WT	WT
1 (2.2)	WT	WT	L374R (AAC → TAC)	WT	WT
1 (2.2)	−15C → T	WT	WT	N111S (AAC → AGC)	WT
1 (2.2)	WT	WT	WT	N111S (AAC → AGC)	S162T (AGC → ATC)
1 (2.2)	WT	WT	WT	WT	L289R (CTT → CGT)
1 (2.2)	−15C → T	WT	WT	WT	G339A (GGG → ATC)
1 (2.2)	WT	WT	WT	WT	S388N (AGC → AAC)
9 (19.6)	WT	WT	WT	WT	WT

*ORF: open reading frame*.

Considering that the genetic mutations in *inhA* confer the cross resistance between INH and PTH, we further sequenced the fragments of *ethA, ndh* and *mshA* in 236 PTH-susceptible isolates to identify the specificity of these novel mutations, which may be associated with PTH resistance. As summarized in Table 6, seven synonymous mutations and two non-synonymous mutations were identified in the 30 (12.7%) PTH-susceptible isolates, while all these mutations were different from the previous non-synonymous SNPs among PTH-resistant isolates.

**Table 6:** Mutations in *ethA, mshA*, and *ndh* in 236 PTH-susceptible MTB isolates.

**Locus**	**Nucleotide change**	**Mutation type**	**Amino acid change**	**No. of isolates (%)**
*ethA*	G → T at 297	Synonymous	R99R	18 (7.6)
	C → T at 342	Synonymous	N114N	2 (0.8)
	A → G at 722	Non-synonymous	K241R	1 (0.4)
	G → A at 879	Synonymous	L293L	1 (0.4)
	T → G at 918	Synonymous	R306R	1 (0.4)
*mshA*	A → G at 174	Synonymous	A58A	1 (0.4)
*ndh*	C → T at 60	Synonymous	I20I	4 (1.7)
	C → A at 135	Synonymous	R45R	1 (0.4)
	C → G at 533	Non-synonymous	A178G	1 (0.4)

### Association of the MTB sublineages with genetic mutations

Among 34 Beijing genotype strains classified as PTH-resistant, a total of 28 isolates (82.4%) carried the genetic mutations within five candidate genes conferring PTH resistance, including 20 (58.8%) with single mutation and 8 (23.5%) with double mutations. 9 (75.0%) out of 12 non-Beijing genotype strains had nucleotide substitutions. Statistical analysis revealed that no significant difference was found in the distribution of isolates with different mutation types between Beijing and non-Beijing genotypes (*P* > 0.05). In addition, we further analyzed the prevalence of isolates with mutations at different loci between Beijing and non-Beijing genotypes, while there was also no statistical difference among five candidate genes between the groups (*P* > 0.05; Table 7).

**Table 7:** Distribution of different mutations conferring PTH resistance among Beijing and non-Beijing genotypes.

**Mutation type**	**No. of PTH-resistant isolates with different mutations (%)**						
	**Beijing (*****n*** = **34)**				**Non-Beijing (*n* = 12)**	***P*-value**	**Total (*n* = 46)**
	**Ancient (*n* = 12)**	**Modern (*n* = 22)**	***P*-value**	**Total**			
**LOCUS**							
*inhA* promoter	6 (50.0)	7 (31.8)	0.46	13 (38.2)	3 (25.0)	0.50	16 (34.8)
*inhA* ORF	1 (8.3)	3 (13.6)	1.00	4 (11.8)	2 (16.7)	0.64	6 (13.0)
*ethA*	5 (41.7)	10 (45.5)	0.83	15 (44.1)	4 (33.3)	0.74	19 (41.3)
*mshA*	1 (8.3)	1 (4.5)	1.00	2 (5.9)	0 (0.0)	1.00	2 (4.3)
*ndh*	0 (0.0)	2 (9.1)	0.53	2 (5.9)	2 (16.7)	0.27	4 (8.7)
Single mutation	7 (58.3)	13 (59.1)	1.00	20 (58.8)	7 (58.3)	1.00	27 (58.7)
Double mutation	3 (25.0)	5 (22.7)	1.00	8 (23.5)	2 (16.7)	1.00	10 (21.7)
No mutation	2 (16.7)	4 (18.2)	1.00	6 (17.6)	3 (25.0)	0.68	9 (19.6)

*ORF, open reading frame*.

## Discussion

To our best knowledge, this study was the first to investigate the phenotypic and genotypic characteristics of PTH resistance in China. On the basis of our data, high diversity of genetic mutations conferring PTH resistance is identified among MTB isolates from southern China. The chromosomal mutations in the *ethA* gene remain the major mechanism for PTH resistance, accounting for 41.3% of PTH-resistant isolates of this report, while the frequency was lower than previous studies that 54.2% to 100% of ETH-resistant MDR-TB isolates harboring mutations in this gene (Boonaiam et al., 2010; Rueda et al., 2015). Notably, the genetic mutations in the *ethA* gene are distributed across the structural gene, and no predominant nucleotide substitution is observed among various mutant types (Rueda et al., 2015). The distribution is unlike those observed for markers conferring other antimicrobial agent resistance, for which the mutations located in a single or a limited area of target genes (Köser et al., 2015). In addition to *ethA*, the genome of MTB probably contains about 30 monooxygenases (Baulard et al., 2000). Although the mutation in *ethA* yields a defective protein, the other monooxygenases may compensate its loss of fitness. Thus, we speculate that this diversity in the *ethA* gene may be that mutations confer low to no fitness cost, which enhances the strains to survive under selective pressure. In addition, we also found that mutations in the promoter region of *inhA* and *inhA gene* in PTH-susceptible isolates were different from those in PTH-resistant isolates, and these mutations are more likely to appear together with other mutations in PTH-resistant isolates. Although the exact reason is still not clear, we speculate that these mutations may show cumulative effect on increasing MIC-values of MTB against PTH.

Beijing genotype strains could be further divided into ancient and modern sublineages based on the absence or presence of an IS6110 insertion in NTF region (Zhang et al., 2015). Several lines of research support the notion that these two sublineages undergo different mechanisms of co-existence with human populations (Gagneux et al., 2006; Yang et al., 2012; Zhang et al., 2015). Ancient Beijing strains prefer the higher frequency of drug resistance, while modern Beijing strains depend on the increasing availability of susceptible hosts (Zhang et al., 2015). In line to this hypothesis, we found that ancient Beijing genotype strains were more like to acquire INH and SM resistance compared with modern Beijing strains. The tendency of the ancient Beijing strains to acquire drug resistance has also been revealed in several reports from East Asian areas with high prevalence of Beijing genotype (Mokrousov et al., 2006; Iwamoto et al., 2008; Maeda et al., 2014; Zhang et al., 2015). However, the drug resistance profiles of ancient Beijing strains were different from our observation: INH and RMP in one report (Iwamoto et al., 2008) and KAN in another report (Zhang et al., 2015). It would be interesting to investigate the potential reasons responsible for these differences. On one hand, the underlying mechanism for our observation may be relevant to the history of the antibiotics introduced in the treatment of TB. As the oldest two drugs used for TB treatment in China, the accumulated SM- and INH-resistance during the past decades may explain the correlation between ancient Beijing genotype and resistance to these two drugs. On the other hand, the difference may be also due to the variance in sampling of TB strains. In the latter study, the higher frequency of KAN resistance in ancient strains was found in the MDR-TB strains (Zhang et al., 2015). After harboring RIF and INH resistance, the selective pressure of MDR-TB comes from the application of second-line anti-TB drugs, especially fluoroquinolones (FQs) and second-line injectable drugs. Hence, the relative long period of KAN implemented for TB treatment may partly explain the current spread of KAN-resistant MDR-TB strains.

Another interesting finding of our report was that PTH resistance was more likely to be observed in the LFX-resistant MTB isolates. Similar to our observations, Alame-Emane and colleagues have demonstrated that pyrazinamide resistance in MTB arises after FQ resistance (Alame-Emane et al., 2015). Although the exact reason for this correlation remains unknown at this stage, the higher occurrence of PTH-resistance after FQ-resistance may be due to the production of oxygen radicals induced by the exposure to FQ, and thus increases mutagenesis (Baharoglu and Mazel, 2011). Compared with other anti-TB drugs, the mutant genotypes conferring PTH-resistance are more diverse at numerous loci, thereby resulting in higher frequency of resistance to PTH in MTB. Notably, there is strong evidence that the abuse and overuse of FQs is routinely used for the empirical treatment of numerous outpatient infections, which is responsible for the increasing emergence of FQ-resistant MTB in China (Zhang et al., 2014a). On basis of our findings, the high prevalence of FQ-resistance may lead to the subsequent PTH-resistance in MTB, and thus could reduce the efficacy of regimen containing PTH for the treatment of FQ-resistant patients.

This study has some obvious limitations. First, although BD Bactec MGIT 960 is recommended as the gold standard for detecting susceptibility to second-line anti-TB drugs of MTB isolates (WHO, 2008), the poor reliability and reproducibility have been noted in PTH testing compared with other second-line drugs by several studies (Krüüner et al., 2006; Rüsch-Gerdes et al., 2006; van Ingen et al., 2010). Hence, the unfavorable performance of MGIT 960 for determining PTH susceptibility may affect the correlation between genotype and *in vitro* susceptibility to PTH in MTB isolates. Second, all the isolates enrolled in this study were from a TB specialized hospital, the sampling bias could be associated with the high prevalence of drug-resistant TB in this study. Third, because the majority of Lineage 2 MTB strains are Beijing genotype in China, RD105 was used to differentiate Beijing and non-Beijing genotype strains in this study, as well as several previous reports from eastern Asia (Chen et al., 2008; Zhang et al., 2015). However, a recent study has revealed RD105 is less specific to Beijing genotype compared with RD207 (Ribeiro et al., 2014). This limitation of methodology would confound genotyping analysis. Nevertheless, this study provides the first insights into the molecular characteristic of PTH-resistant MTB isolates in China, which will be essential for developing appropriate molecular diagnostic assays for the detection of PTH susceptibility of MTB in China.

## Conclusion

In conclusion, our data demonstrate that high diversity of genetic mutations conferring PTH resistance is identified among MTB isolates from southern China. The most common mechanism responsible for PTH resistance has been traced to mutations in *ethA* gene. Double mutations seem more frequent than single mutation in PTH-resistant isolates. Ancient Beijing genotype strains have higher proportion of drug resistance compared with modern Beijing strains. In addition, PTH resistance is more likely to be observed in the LFX-resistant MTB isolates. Further studies are needed to confirm the role of *mshA* mutation for PTH resistance in MTB.

## Author contributions

YT and YP: designed this study; YT, BS, HZ, YS, YW, and YP: performed experiments; YT and YP: interpreted the data: YT, BS, and YP: wrote the manuscript. All authors approved the final version of the paper.

### Conflict of interest statement

The authors declare that the research was conducted in the absence of any commercial or financial relationships that could be construed as a potential conflict of interest.
