# Supplementary figures and images for: Molecular Characterization of Prothionamide-Resistant Mycobacterium tuberculosis Isolates in Southern China
Source: Front Microbiol. 2017 Nov 30;8:2358. doi: 10.3389/fmicb.2017.02358 (PMC5714880; doi:10.3389/fmicb.2017.02358)

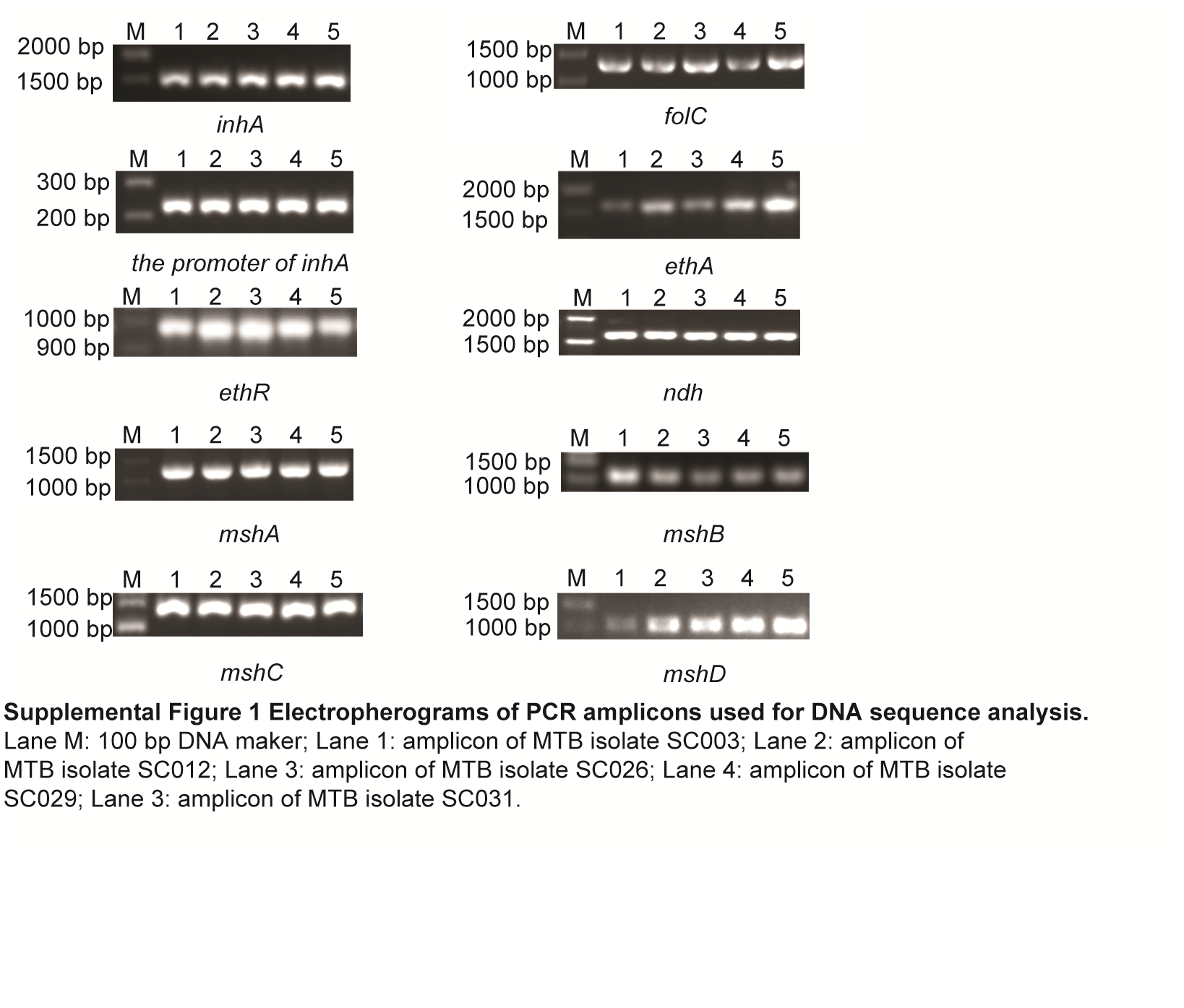

Supplement: Supplementary file 1 [file Image1.TIF]
